# Role of PI3K/Akt/GSK-3 Pathway in Emesis and Potential New Antiemetics

**DOI:** 10.33696/Signaling.1.024

**Published:** 2020-12

**Authors:** W Zhong, NA Darmani

**Affiliations:** Department of Basic Medical Sciences, College of Osteopathic Medicine of the Pacific, Western University of Health Sciences, 309 East Second Street, Pomona, CA 91766, USA

**Keywords:** Chemotherapy, Emesis, Ca2+, Emetic nuclei, PI3K, Akt, GSK-3

Nausea and vomiting are protective defense mechanisms by which vomit competent species avoid ingestion of potentially toxic substances. More specifically, vomiting is the act of forceful expulsion of gastrointestinal contents through the mouth, whereas nausea is an unpleasant painless subjective feeling that one will imminently vomit. Severe or chronic vomiting can become detrimental due to significant loss of fluid and ion imbalance. The act of vomiting is usually preceded by retching, where the gastrointestinal tract contents are forced into the esophagus, without the vomitus being expelled [1]. While significant knowledge exists on the neurotransmitter and anatomical basis of vomiting [2-4], nausea is the neglected symptom and its anatomical neurochemistry remains to be fully defined.

The major emetic sites involved in the process of vomiting include: i) the brainstem dorsal vagal complex (DVC) containing the central emetic nuclei such as the area postrema (AP), nucleus tractus solitarius (NTS) and dorsal motor nucleus of the vagus (DMNX); and ii) the peripheral emetic loci such as neurons of the enteric nervous system (ENS) and enterochromaffin cells (EC cells), as well as vagal afferents carrying input from the gastrointestinal tract (GIT) to the brainstem DVC [5,6]. Cisplatin-like cancer chemotherapeutics cause vomiting via release of multiple neurotransmitters [e.g. dopamine, serotonin (5-HT), substance P, etc] from the EC cells and/or the brainstem [7]. In the past, non-specific emetogens such as copper sulfate or cisplatin were often used to determine the antiemetic potential of drugs in relatively large animal models of vomiting including dogs, cats, or ferrets [8]. Recently, more specific emetogens are frequently used in emesis research using smaller vomit-competent-species such as least shrews (*Cryptotis parva*) [9] or house musk shrews (Suncus murinus) [10]. Such receptor-selective or non-selective specific emetogens include agonists of serotonin type 3 (5-HT_3_R) (e.g. 2-Methyl-5-HT or 5-HT)-, substance P neurokinin type 1 (NK_1_R) (e.g. GR73632)-, dopamine D_2_ (D_2_R) (e.g. quinpirole or apomorphine)-, and muscarinic 1 (M_1_R) (McN-A-343 or pilocarpine)-receptors, as well as Ca^2+^ channel regulators comprising the L-type Ca^2+^ channel (LTCC) agonist FPL64t76 [11], and the sarco/endoplasmic reticulum Ca^2+^-ATPase (SERCA) inhibitor thapsigargin [12]. Based on our Ca^2+^-dependent emesis hypothesis [9], we have demonstrated the broad-spectrum antiemetic nature of two of the selective LTCC inhibitors, nifedipine and amlodipine, against the above discussed diverse emetogens [11-13].

In this laboratory we have focused on investigating intracellular emetic signals evoked by the above discussed specific emetogens. Indeed, our recent findings have well established that ERK1/2 is a common emetic signal in the mediation of vomiting elicited by intraperitoneal administration of diverse emetogens [12,14-18]. Moreover, our group has demonstrated a time-dependent upregulation of phosphorylation of protein kinase B (Akt) downstream of phosphoinositide 3-kinase (PI3K) signaling in the least shrew brainstem following administration of either the selective LTCC agonist FPL64176 [19] or the emetic NK_1_R agonist GR73632 [14]. Following PI3K activation, phosphatidylinositol (3,4,5)-trisphosphate (PIP3) accumulates at the cell membrane which then leads to the recruitment of Akt to the plasma membrane where Akt is phosphorylated at Thr308 together with Ser473 which ensures full Akt activation [20,21]. Multiple cellular experiments have shown that the PI3K inhibitor LY-294002 can inhibit the activity of its downstream target protein, Akt, therefore it is more often described as a PI3K/Akt inhibitor [20-22]. In a recent study we found that LY-294002 at 20 mg/kg (i.p.) dose, could reduce both: i) the vomiting evoked by the neurokinin NK_1_R selective agonist GR73632 in least shrews, and ii) the GR73632-evoked ERK1/2 phosphorylation and Akt phosphorylation at Ser473 in the shrew brainstem protein extracts. These findings suggest an important role for the PI3K-Akt pathway in NK_1_R-mediated emesis [14]. However, the role of Akt in the evoked vomiting appears to be complex and is under continued investigation. Indeed, our recent unpublished findings indicate that PI3K/Akt pathway inhibitors are potent emetogens in the least shrews when administered systematically, which we further discuss in the following paragraph.

The PI3K/Akt pathway hyperactivation occurs in several types of cancers and inhibitors targeting this pathway are under development as potential armamentarium for cancer treatment which have been extensively reviewed [23,24]. When treating cancer patients with PI3K/Akt pathway inhibitors, nausea and vomiting are among their common impending side-effects [25]. Indeed, GSK2636771, the potent, orally bioavailable, adenosine triphosphate-competitive and selective inhibitor of PI3Kβ, not only causes dose-dependent nausea (40%) and vomiting (31%) in cancer patients, but also reduces the phospho/total Akt ratio [26]. In our one of our current animal studies, we observed that a larger dose of the PI3K-Akt inhibitor LY-294002 (40 mg/kg., i.p.) caused vomiting in up to 71% of tested shrews, whereas its lower dose (20 mg/kg) has limited emetic potential. Such anti/proemetic effect with small versus larger doses of other antiemetics, such as the selective 5-HT_3_ receptor antagonist tropisetron, has also been observed against GR73632-evoked NK_1_ receptor-mediated emesis [27]. In addition, Akt inhibitors, perifosine and MK-2206, appear to be more efficacious proemetic than LY-294002. In fact, clinical trials demonstrate that perifosine evokes vomiting in up to 63% of cancer patients [28]. Likewise, relative to perifosine, MK-2206 is a more potent and efficacious proemetic chemotherapeutic in cancer patients [29], which mirrors the findings of our studies in least shrews. In fact, while MK-2206 induced vomiting in all tested least shrews at 10 mg/kg (i.p.) dose, perifosine evoked emesis in ~90% of tested shrews at 50 mg/kg (i.p.). Moreover, MK-2206-evoked emesis led to a significant increase in c-Fos expression in the shrew brainstem DVC emetic nuclei (AP, NTS and DMNX) as well as in the intestinal jejunum ENS. The latter findings support the notion that the MK-2206-evoked vomiting is mediated via activation of both central and peripheral emetic loci. Consistent with our previous findings [12,14], the Akt inhibitor MK-2206 evoked significant ERK1/2 phosphorylation in both the brainstem DVC emetic nuclei as well as the jejunal ENS. The selective ERK1/2 inhibitor U0126 (10 mg/kg, i.p.) prevented MK-2206-induced vomiting, suggesting ERK1/2 signaling contributes to emesis in response to pharmacological inhibition of Akt. Our finding is in line with several published studies that demonstrate PI3K/Akt inhibition leads to the RAF/MEK/ERK pathway activation. Indeed, PI3K inhibitors wortmannin or LY-294002 as well as Akt inhibitor VIII, significantly potentiate ERK1/2 phosphorylation at the cellular level [30]. Moreover, upregulation of phosphorylated ERK1/2 has also been observed following exposure to MK-2206 [31,32], which is consistent with our findings.

Based on the essential role of intracellular Ca^2+^ mobilization in the process of vomiting evoked by diverse emetogens [9], we evaluated the antiemetic potential of the LTCC blocker nifedipine against MK-2206- and perifosine-induced vomiting. Nifedipine provided potent and total (100%) protection against the vomiting caused by both Akt inhibitors perifosine (50 mg/kg, i.p.) and MK-2206 (10 mg/kg, i.p.). At this dose nifedipine could only partially protect shrews from vomiting evoked by the above discussed diverse emetogens, including that caused by LTCC activator FPL64176 [11]. Thus, Akt inhibitor-evoked emesis is highly sensitive to the LTCC blocker nifedipine, and extracellular Ca^2+^ entry through LTCCs plays a major role in both perifosine- and MK-2206- induced emesis. Our preliminary results support the involvement of serotonergic 5-HT_3_Rs, neurokinin NK_1_Rs and dopamine D_2_Rs in vomiting evoked by the Akt inhibitor MK-2206. In fact, according to the antiemetic efficacy profiles of the selective 5-HT_3_ receptor antagonist palonosetron, NK_1_R antagonist netupitant, and the selective D_2_R antagonist sulpiride, we propose that release of emetic neurotransmitters serotonin, substance P and dopamine are involved in MK-2206-evoked emesis. Further studies are to be performed in our laboratory to address their underlying mechanisms.

A downstream target protein for Akt signaling pathway, glycogen synthase kinase-3 (GSK-3), is a constitutively active protein involved in diverse physiological processes including metabolism, cell cycle, and gene expression [33-38]. GSK-3 is also involved in a wide range of pathologies such as diabetes, inflammation, some types of cancer, neurodegeneration and mental illness [39]. GSK-3 is encoded by two known genes, GSK-3α and GSK-3β. Activation of Akt signaling can be followed by phosphorylation of GSK-3α/β at Ser21/9 and its subsequent inactivation [35]. Interestingly, in another study [40], we have demonstrated that GSK-3 also plays a role in vomiting. Phosphorylation of GSK-3α and GSK-3β subtypes in the least shrew brainstem displays a time-dependent increase in response to a variety of emetogens, including agonists of serotonin 5-HT_3_ (e.g. 5-HT or 2-Methyl-5-HT)-, neurokinin NK_1_ (GR73632)-, dopamine D_2_ (apomorphine or quinpirole)-, muscarinic M_1_ (MCN-A343 or pilocarpine)-receptors, as well as the LTCC agonist FPL64176 and the SERCA inhibitor thapsigargin [40]. Moreover, immunostaining has confirmed that GSK-3α/β phosphorylation at Ser21/9 exhibit increased immunoreactivity in the least shrew brainstem DVC emetic nuclei (AP, NTS and DMNX) in response to cisplatin administration (10 mg/kg., i.p.) [40]. Following administration of diverse emetogens including cisplatin, an increase in both phospho-GSK-3α Ser21 and phospho-GSK-3β Ser9 was also observed in the jejunal ENS of least shrew at 5 h post cisplatin administration (10 mg/kg., i.p.) [40].

Functionally, the GSK-3 inhibitor AR-A014418, has been shown to dose-dependently suppress both the frequency and percentage of shrews vomiting evoked by the above specific emetogens. Another GSK-3 inhibitor SB216763 is a more potent antiemetic than AR-A014418, since at lower dosage it exerted such broad antiemetic efficacy [40]. Differences in pharmacological properties of the two tested GSK-3 inhibitors may contribute to their differential antiemetic potential. Indeed, AR-A014418 is a selective ATP-competitive GSK-3β inhibitor (IC_50_ = 104 nM) [36, 41-43], whereas SB216763 is considered as a more potent but equally effective inhibitor of both GSK-3α and GSK-3β (IC_50_ = 34.3 nM) [44,45]. These data support an interesting hypothesis that following pharmacological induction of vomiting, the evoked phosphorylation (i.e. inhibition) of GSK-3 may exert a unique self-protective effect to avoid further vomiting. Thus, utilization of GSK-3 inhibitors may become a useful strategy for achieving a greater degree of inhibition of GSK-3 activity for suppression of vomiting.

In addition, we co-stained the least shrew jejunal sections with phospho-GSK-3α Ser21 with a 5-HT antibody (generally accepted as a marker of the intestinal enterochromaffin (EC) cells). The attained results indicate significant presence of phospho-GSK-3α Ser21 in EC cells, while phospho-GSK-3β Ser9 was barely detected in these phospho-GSK-3α Ser21 positive EC cells. In this regard, it would be extremely important to design future experiments with more highly selective and potent GSK-3α and GSK-3β paralogs so that their corresponding antiemetic potential and matching signaling pathways could be further assessed both in the brainstem and the intestine.

Another downstream target protein for the PI3K/Akt signaling pathway is the mammalian target of rapamycin (mTOR), which is a master regulator of cellular metabolism, plays a central role in the regulation of autophagy and a pivotal role in cancer [46]. Our recent unpublished data shows varying doses of mTOR inhibitor rapamycin (0, 5, and 10 mg/kg., i.p.) induces vomiting in the least shrews in a dose-dependent manner with a maximal effect at 10 mg/kg (i.p.). The mechanisms of the emesis induced by pharmacological inhibition of the multifaceted mTOR is under investigation in our lab.

In summary, chemotherapy-induced nausea and vomiting affects the well-being and quality of life of cancer patients receiving chemotherapy. Selective serotonin 5-HT_3_ antagonists combined with neurokinin NK_1_ antagonists are at present the most effective therapeutic agents [3,4]. In this commentary, the discussed findings from the least shrew emesis model helps to open new avenues for antiemetic research against chemotherapeutic agents, as well as infectious agents including viruses and bacterial toxins [47]. These findings should be further tested in other established animal models of vomiting as well as in patients. The potential targets could include not only Ca^2+^ signals but also other signaling pathways such as ERK1/2 and PI3K/Akt with downstream GSK-3 and mTOR.
